# Observation of diffraction contrast in scanning helium microscopy

**DOI:** 10.1038/s41598-020-58704-1

**Published:** 2020-02-06

**Authors:** M. Bergin, S. M. Lambrick, H. Sleath, D. J. Ward, J. Ellis, A. P. Jardine

**Affiliations:** The Cavendish Laboratory, JJ Thomson Avenue, Cambridge, CB3 0HE UK

**Keywords:** Condensed-matter physics, Imaging techniques, Atomic and molecular collision processes

## Abstract

Scanning helium microscopy is an emerging form of microscopy using thermal energy neutral helium atoms as the probe particle. The very low energy combined with lack of charge gives the technique great potential for studying delicate systems, and the possibility of several new forms of contrast. To date, neutral helium images have been dominated by topographic contrast, relating to the height and angle of the surface. Here we present data showing contrast resulting from specular reflection and diffraction of helium atoms from an atomic lattice of lithium fluoride. The signature for diffraction is evident by varying the scattering angle and observing sharp features in the scattered distribution. The data indicates the viability of the approach for imaging with diffraction contrast and suggests application to a wide variety of other locally crystalline materials.

## Introduction

The ability to image the surfaces of materials is fundamentally important to many scientific disciplines, and historically, the development of new forms of microscopy have underpinned the development of broad areas of research. However the ability to image certain materials, including those sensitive to charge, or to the energy of the probe particles, remain a particular challenge. Recently, scanning helium microscopy (SHeM) has emerged as a new tool for measuring delicate materials, making use of extremely low energy neutral atoms to form images^1–3^. New forms of contrast are possible without any concern of beam induced damage to the sample.

The SHeM technique involves producing a thermal energy beam of helium atoms (typically 5–100 meV), then collimating or focussing the beam to form a microprobe, which is rastered across the surface of a sample. Atoms scattered in a particular direction from the illuminated spot on the surface are counted and used to form the scattered intensity of each pixel in the image. The approach was originally demonstrated in transmission by Koch *et al*.^1^ and was later extended to reflection imaging by Witham and Sanchez^3,4^ and collaborating researchers in Cambridge, UK and Newcastle, Australia who used pinhole collimation^5^.

As the field of helium microscopy develops, it is vitally important to explore and understand the possible origins of contrast in helium images. The topic was first discussed by MacLaren *et al*.^6^, and there has been considerable recent interest^2,7,8^. In general, contrast in helium images arises as a result of changes in the angular distribution of scattered helium atoms with location on the surface. Helium atom scattering (HAS) has long been used to study the properties of surfaces under high and ultra-high vacuum conditions, so the basic helium– surface interaction mechanisms are well understood^9–12^. For surfaces that are well defined at an atomic level, incoming helium atoms can reflect specularly when the surface appears flat, diffract from atomic corrugation, or interact inelastically with surface phonons in the material.

Since helium atoms scatter from the outermost electrons in a surface, HAS is extremely sensitive to any form of surface contamination – and in particular under ambient conditions most ‘clean’ surfaces will quickly contaminate. To date, almost all SHeM images show contrast consistent with diffuse scattering combined with topographic variations in the surface profile^3,7,13^ – i.e. at an atomic level the surface appears disordered to the helium beam. Specifically, the diffusely scattered distribution is broad and centred approximately on the surface normal, so is consistent with Knudsen’s empirical law^14^ or alternatively Lambert’s cosine law for photons. Such contrast has become referred to as ‘topographic contrast’ in recent literature. Other forms of contrast have been suggested or tentatively observed, including chemical contrast^2^ and Debye– Waller contrast^6^.

In this paper, we report the first helium images showing contrast arising from specular reflection and diffraction at an atomically clean surface. We describe helium images of a sample of lithium fluoride (LiF), cleaved along the approximate 〈100〉 plane. LiF was chosen as when cleaved, it known to readily provide an inert, atomically flat surface that is capable of diffracting helium^15,16^. We present images that show structure due to imperfect cleavage which can be associated with the usual topographic contrast. In addition, there are areas of strong local intensity enhancement, which indicate specular and diffraction effects. The origin of the enhancement was confirmed by performing spot-profile measurements; varying the position of the sample relative to the specular scattering condition enables individual diffraction peaks to be directly observed within the SHeM.

Our results demonstrate the feasibility of using diffraction contrast, thus enabling experiments to be designed that utilise the new contrast mechanism. We expect the approach to be of significant value in imaging any well defined locally crystalline surfaces, in particular delicate polycrystalline materials such as organic thin-films, and could significantly broaden the appeal of the SHeM technique. Diffraction contrast could also find broader application by using beams other than helium, since it has been shown that many other species (for example H_2_ or D_2_^17,18^) can diffract strongly at surfaces. The remainder of the paper is organised as follows: it begins with an overview of the Cambridge SHeM with a particular focus on the approach we have used to enable diffractive contrast to be conclusively identified. Next a numerical simulation is presented to establish a clear signature for diffraction contrast in the SHeM. Finally, a series of images for LiF showing contrast enhancement is provided, followed by experimental spot-profiles to conclusively determine the origin of the effect.

## Contrast Formation in the Cambridge SHeM

**Figure 1:**
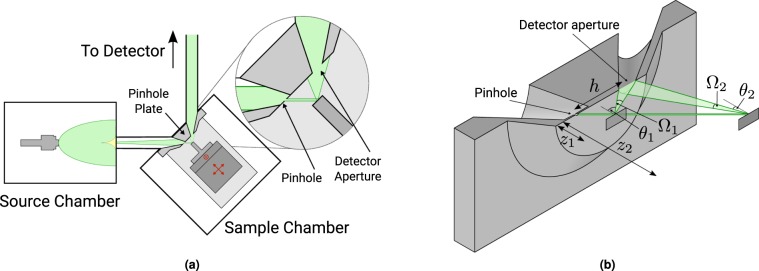
(**a**) Overview of the Cambridge scanning helium microscope. The instrument consists of a helium beam source and differential pumping stage, after which the beam is collimated using a pinhole aperture. The microprobe is incident on a sample, and scattered atoms are transferred to a high sensitivity detector. The pinhole plate defines the way in which contrast is generated. (**b**) Schematic showing the pinhole plate and how the sample is moved during a *z* scan, including those variables that are changed as the pinhole to sample distance is increased. Since the incoming beam is oriented at 45° to normal, when the sample moves in *z* it also needs to be shifted laterally to ensure that the same part of the surface is kept illuminated. When the sample to pinhole distance is changed, both the outgoing angle of the gas that is detected and the solid angle of the detector aperture also change.

A schematic overview of the Cambridge SHeM is shown in Fig. 1a. The instrument consists of a helium beam source (left), where high pressure helium (~100 bar) is expanded through a fine nozzle (10 μm) into vacuum. A 100 μm skimmer is used to extract the centreline of the expanding gas, to form a beam. The beam is passed through a differential pumping stage to reduce the background gas pressure, then is further collimated by a pinhole (1.2 μm diameter in the present measurements) to form the helium microprobe. The microprobe is incident on the sample at an angle of 45° to the average surface normal. Atoms are then scattered at the sample and some pass through a collection aperture at a nominal 90° total scattering angle, which are transferred to a high sensitivity helium detector for counting^19,20^. The image is constructed by rastering the sample position.

The geometry of the sample environment, shown in more detail in Fig. 1b is crucial to determining the form of contrast produced. The key parameters are the scattering angles, the solid angle of the detection aperture, Ω, the pinhole to sample working distance, *z*, and the distance between the pinhole and the detector aperture, *h*. In the current instrument the apertures and angles are primarily defined by the ‘pinhole plate’ which includes both the pinhole and detection apertures. By varying the distance between the sample and the pinhole, *z*, it is possible to measure the scattered intensity at different angles. Given the geometry, in order to illuminate the same point on the sample while varying *z*, the sample also needs to be moved laterally. We refer to varying *z*, while keeping the same point on the sample under the beam, as a *z* scan. A complication that arises when performing a *z* scan is that the size of the beam changes as the distance between the sample and the pinhole is varied^21,22^. The range of scattering angles that can be observed is therefore determined by both the available signal level and the largest acceptable illumination area of the helium beam, since resolution of a small feature would require a narrow helium beam.

**Figure 2:**
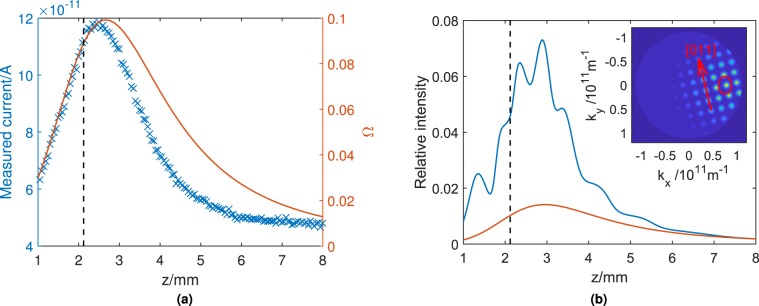
(**a**) Plot of a *z* scan taken from a sample exhibiting diffuse scattering (highly oriented pyrolytic graphite, blue points) and the change in detector aperture solid angle (orange curve) during a *z* scan, calculated using equ. 52 from Conway^27^. The specular condition is labelled with a dashed line. As the distance between the sample and pinhole is varied, the majority of the change in the detected flux is due to the change in solid angle of the detector aperture. The remaining variation is due to the change in transmission probability through the detector cone. (**b**) Simulated *z* scan on LiF using the simplified diffraction model described in the text and shown in the inset, to illustrate the general form expected with diffractive scattering. The orange curve illustrates the diffuse component of the scattered helium, while the blue curve illustrates the total scattered beam including both the diffractive and diffuse components. The peaks in the *z* scan correspond to different diffraction orders entering the detector aperture. The specular condition is labelled with a dashed line. (Inset) shows the representative diffraction pattern for a helium beam at 298 K with 45° incidence. The lattice is rotated anticlockwise by 14° to match the experimental setup described later. Note that diffraction peak heights are not calculated rigorously, and simply reduce in intensity around the specular beam. The large detector aperture (shown as a red circle) leads to the possibility of multiple peaks entering the detector at once.

In the case of topographic contrast from a rough surface, atoms are scattered in many directions in accordance with the local topography across the surface area covered by the beam, thus (excluding shadowing or masking effects) a fraction can always reach the detector. Contrast primarily arises from differences in the local angle of the surface, which affects the number of atoms passing into the detector through a wide diffuse scattering distribution, centred approximately around the surface normal. When the working distance increases, the apparent solid angle of the detector changes, as does the probability of atoms being transmitted through the cone section. The latter is due to the angle with which scattered atoms enter the detector aperture; atoms at large angles quickly hit the walls of the cone and are therefore more likely to be scattered out of the cone again, rather than reaching the detector^7^. Both these factors affect the probability of atoms being detected and result in a peaked, but otherwise smoothly varying detection function with distance. Figure 2a shows a typical *z* scan from a rough surface (HOPG has been chosen as a representative example of diffuse scattering), indicating the general variation expected under such circumstances. The distribution therefore causes a height-based contrast mechanism, but which only introduces useful contrast changes over rather large height differences, of order 100 μm or more. It also explains the origin of the recently observed contrast inversion with *z*^8^.

If a surface scatters specularly or diffracts the incoming helium atoms, the angular distribution of scattered atoms consists of well defined diffraction peaks. The fact that our instrument has a total scattering geometry of 90° means that at the correct *z* distance, the specular reflection from a flat surface (if present) can be transferred directly into the detector, unlike in the case of a normal incidence apparatus^4^. Note that the specular reflection does not necessarily occur at precisely the same point as the maximum in Fig. 2a. At other *z* positions, diffraction peaks can enter the detector, depending on the azimuthal orientation and spacing of the local crystal lattice. Compared to traditional atom scattering instruments, the low angular resolution of the SHeM means that at any given working distance at least one such peak is likely to contribute to the detected signal. As we will show below, the key signature for diffraction effects to be contributing to contrast is thus the superposition of steps or peaks onto the otherwise smooth variation in signal within a *z* scan, *i.e*. peaks appearing superimposed on the variation shown in Fig. 2a.

## Diffraction Simulation

The scattering distribution at a particular surface location can be measured directly in the microscope by performing a *z* scan. However, the solid angle of scattered helium that is collected for detection is relatively large and varies throughout a scan, complicating interpretation and making the expected form of the scan rather non-intuitive. For comparison with experiments, it is therefore useful to make a semi-quantitative prediction of the general form expected. We consider the surface of LiF, a surface that has long been used in the atom-surface scattering community^23^ as a model for diffraction, and which we measure experimentally below. There have been many studies of helium diffraction from LiF^15,16^. However, there is no published two dimensional diffraction data measured under the conditions relevant to the microscope. Predicting the full diffraction pattern using computational methods remains a significant challenge, but fortunately the peak locations can be identified using less complex methods. We can therefore illustrate the general form with which diffraction contrast would appear in a *z* scan, without having to calculate the exact signal that would be expected - i.e. simulated peak locations will be correct, although their heights and widths will not.

Firstly, the locations of the diffraction peaks need to be determined. LiF has an f.c.c. lattice, and the fluoride ions in the (100) plane have a simple cubic structure with a spacing of 2.84 Å^16^. Assuming that the corrugation in the electron density is dominated by the positions of the fluoride ions, the diffraction peak positions are then given by,

1 $${{\bf{K}}}_{{\bf{f}}}={{\bf{K}}}_{{\bf{i}}}+{\bf{G}},$$

where **K**_**i**_ and **K**_**f**_ are the two dimensional wavevectors of the incoming and scattered helium, parallel to the surface, and **G** is a two dimensional surface reciprocal lattice vector. An approximation to the diffraction pattern was produced by placing Gaussian peaks at each of the diffraction peak locations with widths chosen to approximately match the widths of the peaks measured by Boato *et al*.^15^ (standard deviation of 3.5 × 10^9^ m^−1^). The heights of the peaks were also given a Gaussian distribution around the specular, with the peaks decaying such that, again, the diffraction pattern is consistent with data measured by Boato *et al*.^15^ (the second order peaks are exp (1/2) lower than the specular).

The diffraction peaks were superimposed on a background, to include diffuse scattering due to surface imperfection. Previous work on diffuse scattering^24^ has suggested that the angular distribution is consistent with a cosine distribution. Therefore, the molecular flux, *dn*, in an infinitesimal solid angle, *d*Ω, is assumed to be given by^25^,

2 $$\begin{array}{rcl}dn & = & \frac{1}{\pi }\,\cos (\theta )d\Omega ,\\  & = & \frac{1}{\pi }\,\cos (\theta )\sin (\theta )d\theta d\phi ,\end{array}$$

where *θ* is the polar angle and *ϕ* is the azimuthal angle, as usually defined in spherical polar coordinates and the factor of 1/π is obtained from normalisation. Transforming between representing the scattering using an angular distribution and a reciprocal space distribution requires the change of variables,

3 $${k}_{x}=k\,\sin \,(\theta )\,\cos \,(\phi ),$$

4 $${k}_{y}=k\,\sin \,(\theta )\,\sin \,(\phi ),$$

where *k* is the magnitude of the outgoing wavevector and *k*_*x*_, *k*_*y*_ are the *x* and *y* components of the outgoing wavevector respectively. In the absence of experimental data on the energy of the scattered helium, it is assumed that the diffuse scattering is elastic such the magnitude of the outgoing wavevector is the same as the incoming beam. It can be shown^26^ that the integral to determine the flux, *N*, after a change in variables is given by,

5 $$\begin{array}{rcl}N & = & \mathop{\int }\limits_{R}\,dn,\\  & = & \mathop{\int }\limits_{R}\,{P}_{\theta ,\phi }(\theta ,\phi )\,d\theta d\phi ,\\  & = & \mathop{\int }\limits_{R^{\prime} }\,{P}_{\theta ,\phi }(\theta ,\phi ){|\frac{\partial ({k}_{x},{k}_{y})}{\partial (\theta ,\phi )}|}^{-1}d{k}_{x}d{k}_{y}\end{array}$$

where *R* and *R*′ are the regions representing the detector aperture with (*θ*, *ϕ*) and (*k*_*x*_, *k*_*y*_) respectively and the Jacobian $|\partial ({k}_{x},{k}_{y})/\partial (\theta ,\phi )|$ is given by,

6 $$\begin{array}{rcl}|\frac{\partial ({k}_{x},{k}_{y})}{\partial (\theta ,\phi )}| & = & |\begin{array}{ll}\frac{d{k}_{x}}{d\theta } & \frac{d{k}_{y}}{d\theta }\\ \frac{d{k}_{x}}{d\phi } & \frac{d{k}_{y}}{d\phi }\end{array}|\\  & = & {k}^{2}\,\sin \,(\theta )\,\cos \,(\theta ).\end{array}$$

Combining Eqs. 2, 5 and 6 with the definition $dn={P}_{{k}_{x},{k}_{Y}}({k}_{x},{k}_{y})d{k}_{x}d{k}_{y}$, gives the reciprocal space distribution for diffuse scattering,

7 $${P}_{{k}_{x},{k}_{Y}}({k}_{x},{k}_{y})=\frac{1}{\pi {k}^{2}},$$

which is a constant. The total scattering distribution is then obtained by combining the diffuse and diffractive components, where we choose equal intensities to match the qualitative shape of the experimental data. The result is shown in the inset to Fig. 2b.

To compute the intensity of helium that would be measured in the microscope, the diffraction pattern must be integrated, following the size and direction of the detection aperture. The scattering distribution is projected onto the pinhole plate using,

8 $$x=\left(\frac{{k}_{x}}{{k}_{z}}+1\right){z}_{p},\,y=\left(\frac{{k}_{y}}{{k}_{z}}\right){z}_{p}.$$

The additional term in the *x* position arises due to the horizontal shift that is needed in a *z* scan. The code to implement the calculation is provided on GitHub and Zenodo (10.5281/zenodo.3474164). The validity of the code can be verified by numerically calculating the solid angle of the detector aperture in reciprocal space and comparing it to a known analytic expression. Using the angular distribution for the solid angle, $P{\text{'}}_{\theta ,\phi }(\theta ,\phi )=A\,\sin \,(\theta )$, an excellent agreement was found between the numerical code developed here and the analytical equation for the solid angle given by Conway^27^.

Figure 2b shows a simulated *z* scan, with clear peaks appearing when each diffraction peak moves through the detector aperture. Although the peak heights are not accurate (in fact, it is known that the large corrugation of LiF results in some diffraction peaks being larger than the specular), the general form and spacing are clear. There is no other known mechanism which can result in sharp peaks within a *z* scan, so the peak shape and spacing give an unambiguous signature for diffraction, compared to the smooth variation otherwise expected from diffuse scattering.

Interference between adjacent terraces can be observed in helium scattering by monitoring the intensity of the specular peak while the incoming wavevector is altered either through varying the incidence angle^9,28^ or the beam energy^29^. However, in the experiments described in this paper, the incoming wavevector and beam energy are kept constant, so the concept of monitoring interference oscillations on specular does not apply. As the outgoing angle is varied during a *z* scan, interference can still occur between beams scattered from different terraces. Interference only applies in the case of coherent scattering, thus the effect will only lead to modulation of the diffraction peak intensities. As already discussed, quantitative analysis of diffraction peak intensities is complex and beyond the scope of the present work. However, any such modulation due to terrace interference still requires diffraction from the terraces in the first place, so does not change any of our conclusions.

## Experimental Results

### Sample preparation and image acquisition

A sample of LiF was cleaved along the approximate 〈100〉 plane, mounted on a sample stub, loaded into the Cambridge SHeM and then pumped down to high vacuum within approximately 20 minutes. The pressure in the sample environment was allowed to reach the 10^−8^ mbar range before performing helium imaging, to reduce the possibility of contamination of the high sensitivity detector. No further sample preparation was required or performed.

### Neutral helium images

**Figure 3:**
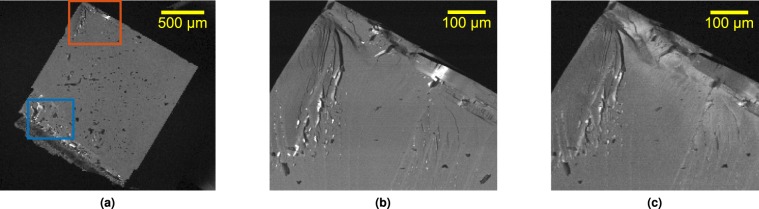
(**a**) SHeM image (208 × 183 pixels) of the cleaved LiF crystal described in the text. The surface is mostly homogeneous with only a few defects, as expected. The lower region of the crystal contains more defects due to imperfect cleaving. Brightness of the top 0.04% of pixels have been clipped. (**b**) SHeM image (400 × 333 pixels) of the top region of the sample, marked by the orange box in (**a**), acquired at *z* = 2.5 mm. Brightness of the top 0.3% of pixels have been clipped. (**c**) SHeM image (220 × 200 pixels) of the same top region of the crystal, but obtained at *z* = 3.3 mm. Brightness of the top 0.01% of pixels have been clipped. The bright features seen in the images shift as the outgoing scattering angle into the detector is varied by altering *z*, indicating they are due to diffraction contrast. Images were obtained using a settle time of 0.5 s and a dwell/accumulation time of 1.125 s for each pixel.

Figure 3(a) shows a large scale neutral helium image covering the whole sample. The shape of the crystal is clearly evident; in the lower left there is damage due to an imperfect cleave, and across the surface there are numerous small defects which we attribute to local surface damage. In various places, notably at the top and lower left, exceptionally bright spots are visible, which we will discuss in more detail.

Figure 3b,c show SHeM images of the upper part of the crystal surface, as marked by an orange rectangle in Fig. 3a, taken at *z* = 2.5 mm and *z* = 3.3 mm, respectively. Parts of the surface that are not flat create contrast due to masking, as previously observed^7,8^. The bright features can be seen to change depending on the value of *z*, and thus the detection angle. For example, in panel (b) there is a bright band on the top right of the image that is no longer present in panel (c). In fact, acquiring many similar images at different values of *z* indicates that the features change continuously with scattering angle (see data pack for animation). The bright features only appear in narrow bands of the outgoing scattering angle, implying sharp features in the scattering distribution, consistent with diffraction. The contrast enhancement therefore provides additional sensitivity to the local gradient and atomic makeup of the surface, well beyond that which is possible with normal topographic variations.

### *z* scans and Discussion

**Figure 4:**
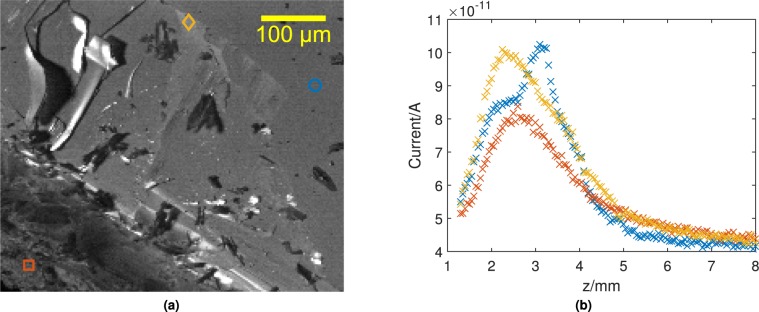
(**a**) SHeM image (212 × 180 pixels) of the lower region in Fig. 3a contained in the blue rectangle, indicating positions where *z* scans were collected. The orange square corresponds to an extremely rough area of the surface, the yellow diamond to a slightly distorted part of the surface, and the blue circle to a flat region. Images were collected using a settle time of 0.5 s and a dwell/accumulation time of 1.125 s for each pixel. Brightness of the top 0.8% of pixels have been clipped. (**b**) Series of *z* scans from the LiF surface at the points indicated in (**a**). The scan from the flat part of the surface (blue) shows a large degree of structure in the scattering distribution, whereas the scan from the distorted region is consistent with the *z* scans more widely on rough surfaces. The largest random noise that was measured on a data point is 1.6 × 10^−13^ A and therefore the error bars have been excluded since they would not be visible.

In order to examine the origin of the contrast enhancement in more detail, a series of *z* scans were taken at selected points on the surface. Figure 4a shows a helium image of the region marked by a blue rectangle in Fig. 3a, and indicates the three points on the surface that were selected for *z* scans. An orange square marks a point in a heavily distorted region of the surface; a yellow diamond indicates a lightly distorted region; and a blue circle is positioned within an apparently flat area. The resulting *z* scans are plotted in Fig. 4b.

The *z* scan for the heavily distorted region (orange) shows a smoothly varying curve comparable with Fig. 2a, following the shape expected for purely topographic contrast from a rough surface. In contrast, the other two curves show significant structure, including narrower peaks. The *z* scan for the flat region, where diffraction is most likely (blue), is most similar to the example in Fig. 2b. The intermediate region (yellow) shows different structure, suggesting diffraction from a different local surface orientation. In both cases the peaks are broader than in the simulation results from Fig. 2b, but are much sharper than the curve expected from purely topographic contrast. The narrower peaks can only arise from coherent scattering, so unambiguously confirm that the origin of the contrast enhancement is surface diffraction.

In principle the diffraction data could be used to infer local surface structure or orientation, although to do so it would be necessary to increase the angular resolution of the measurements. Currently the microscope is configured for topographic imaging, with a relatively large detector entrance aperture to maximize signal (see Fig. 1). For future diffraction contrast measurements, a smaller detector aperture would increase the resolution of individual diffraction peaks, making it easier to investigate the surface properties and would increase the relative fraction of the signal due to diffraction rather than diffuse scattering. A more sophisticated sample manipulation stage with azimuthal rotation would also enable *z* scans to be performed while scattering along the principal directions of the surface lattice.

The helium images and z-scans provide interesting insight into the quality of the cleaved LiF surface. LiF has long been used as a test sample in helium scattering experiments^23,30,31^ yet the origin of the relatively low absolute reflectivity has not been clear. The images show that even an apparently well cleaved surface, when seen by helium atoms, exhibits a large number of small localised defects. Similarly, the *z* scans indicate that there is a substantial diffuse scattering contribution, even in apparently flat areas. The cleaving process also seems to have lifted and deformed certain parts of the surface, such as the bright band shown in Fig. 3b, the local curvature being evident from a series of diffraction images at differing *z* values (see data pack). Given these results, it would be informative to compare the quality of cleavage with different purities of LiF, for future experiments.

## Summary and Outlook

In helium microscopy, nearly all images obtained to date are consistent with diffuse scattering, resulting in topographic contrast. If diffraction also takes place at the surface, the sharp peaks in the scattering distribution result in strong contrast enhancement at the diffraction conditions. The images and analysis presented above conclusively demonstrate that we have made the first observation of diffraction contrast in helium microscopy. Strong contrast enhancement is seen in images of a lithium fluoride surface, and by changing the detection angle (performing *z* scans, as described above), we see diffraction peaks directly, giving an unambiguous confirmation.

We expect that establishing diffraction contrast as a viable imaging approach is likely to significantly increase the applicability of the emerging SHeM technique. In addition to enhancing images, it will enable carefully designed experiments to sensitively probe the atomic makeup of regions of a surface. For example, it would be possible to probe atomic order at the very outermost surface, over very large areas. It could also provide an alternative approach to mapping out the grain structure of complex polycrystalline materials, and might be particularly suited to delicate materials, or thin 2D materials such as graphene. The measurements also form the first step towards an instrument capable of spatially resolved scattering and diffractometry measurements, with a wide range of applicability - effectively bridging the gap between surface science and microscopy with atoms.

## Data Availability

A data pack containing the experimental data presented in this paper is available for download from 10.17863/CAM.47659. The script to perform the simulated *z* scan can be found on Zenodo at 10.5281/zenodo.3474164.
